# Semantic textual similarity for modern standard and dialectal Arabic using transfer learning

**DOI:** 10.1371/journal.pone.0272991

**Published:** 2022-08-11

**Authors:** Mansour Al Sulaiman, Abdullah M. Moussa, Sherif Abdou, Hebah Elgibreen, Mohammed Faisal, Mohsen Rashwan

**Affiliations:** 1 Department of Computer Engineering, College of Computer and Information Sciences (CCIS), King Saud University, Riyadh, Saudi Arabia; 2 Faculty of Computers and Artificial Intelligence, Cairo University, Giza, Egypt; 3 Information Technology Department, College of Computer and Information Science, King Saud University, Riyadh, Saudi Arabia; 4 Center of AI & Robotics, Kuwait College of Science and Technology (KCST), Kuwait City, Kuwait; 5 Center of Smart Robotics Research, College of Computer and Information Science, King Saud University, Riyadh, Saudi Arabia; 6 Artificial Intelligence Center of Advance Studies (Thakaa), King Saud University, Riyadh, Saudi Arabia; 7 Faculty of Engineering, Cairo University, Giza, Egypt; Al-Balqa Applied University Prince Abdullah bin Ghazi Faculty of Information Technology, JORDAN

## Abstract

Semantic Textual Similarity (STS) is the task of identifying the semantic correlation between two sentences of the same or different languages. STS is an important task in natural language processing because it has many applications in different domains such as information retrieval, machine translation, plagiarism detection, document categorization, semantic search, and conversational systems. The availability of STS training and evaluation data resources for some languages such as English has led to good performance systems that achieve above 80% correlation with human judgment. Unfortunately, such required STS data resources are not available for many languages like Arabic. To overcome this challenge, this paper proposes three different approaches to generate effective STS Arabic models. The first one is based on evaluating the use of automatic machine translation for English STS data to Arabic to be used in fine-tuning. The second approach is based on the interleaving of Arabic models with English data resources. The third approach is based on fine-tuning the knowledge distillation-based models to boost their performance in Arabic using a proposed translated dataset. With very limited resources consisting of just a few hundred Arabic STS sentence pairs, we managed to achieve a score of 81% correlation, evaluated using the standard STS 2017 Arabic evaluation set. Also, we managed to extend the Arabic models to process two local dialects, Egyptian (EG) and Saudi Arabian (SA), with a correlation score of 77.5% for EG dialect and 76% for the SA dialect evaluated using dialectal conversion from the same standard STS 2017 Arabic set.

## Introduction

Recognizing the similarity between two sentences is a vital process in many applications since the text is one of the most important media for communication [1]. This makes Semantic Textual Similarity (STS) a critical pre-step in several domains such as information retrieval, document classification, machine translation, textual summarization, question answering, short answer grading, semantic search, and conversational systems [2]. For example, In the information retrieval problem, the most common criterion used to retrieve information is key sentences. Given a set of available media such as documents or videos, millions of them for practical applications, the user can query the system by entering a sentence to describe the content of the medium to be viewed. The same medium can be retrieved using several sentences. i.e., the user can use a different query other than the key sentences that are associated with the medium to describe it. For any efficient retrieval process, the system should be able to recognize the correlation between similar, but different, queries [3].

### STS and sentence embeddings

While there are several ways to tackle the problem of STS, the most promising ones are based on word/sentence embeddings. Sentence embeddings are vector representations of sentences in which each vector is mathematically close in the space to other vectors that represent semantically close meaning. Embeddings can be calculated using different algorithms such as Word2Vec [4], GloVe [5], and BERT [6]. BERT and BERT-Like models are generally based on self-supervised machine learning techniques that make use of the huge amounts of unlabeled text data available on the internet. While BERT is not intentionally created to generate embeddings, it can be adjusted to generate sentence embeddings of good quality. BERT models set new state-of-the-art performance on various sentence classification and sentence-pair regression tasks. To generate a sentence-pair similarity score, BERT uses a cross-encoder: Two sentences are passed to the transformer network and the target value is predicted using a simple regression method for the output. However, this setup is unsuitable for various applications due to the high number of possible combinations to be checked. In [7], the authors proposed a method to generate effective sentence embeddings from BERT models, and several other models have been suggested for such a line of adaptations.

### Motivation

While measuring semantic similarity of texts is applied widely for some languages, for example, English, The Arabic version of the problem has three main limitations. The first one is that the methods proposed to handle the problem for the Arabic language are not of good performance. The second issue is that the development of STS models always requires the availability of semantic similarity annotated corpus with considerable size [8]. Unfortunately, this type of resource is not available for low resources languages such as Arabic. The third problem is that the written form of dialectal Arabic doesn’t have lexical standards. So, there is always a need for approaches that can minimize the gap between the performance of Arabic STS models and the level of STS models of widely investigated languages like English. The motivation of this work is to overcome these challenges. and to provide a methodology for handling these issues. The general advantages and contributions of this work are provided in the next section.

### Contributions

The main contributions proposed in the paper are the following:

- Proposing three approaches to tackle the problem of Arabic STS. The first is to use automatic machine translation to translate English STS data to Arabic and to use the translated data for converting Arabic BERT models into STS Arabic models. The second approach is to interleave English STS data with Arabic BERT models to generate enhanced Arabic STS models. The third approach is based on knowledge distillation models that are optimized using proposed translated Arabic STS datasets.
- The development of a new data resource of professional translation for 1.3K pairs of sentences from their original form in English to MSA, Egyptian Arabic, and Saudi Arabic versions.
- Proposing different models that advance the state-of-the-art performance in the STS task in MSA with limited resources.
- The development, to the best of our knowledge, of first STS models for Egyptian Arabic and Saudi Arabic.

The rest of the paper is organized as follows: Section 2 illustrates the related work and literature review; Section 3 provides the details of the proposed approaches, the developed datasets, and the developed models. Section 4 includes the experimental results and Section 5 includes comparisons with the state-of-the-art results. Finally, section 6 includes the conclusions and some prospects for our planned future work.

## Related work

### Lexical-based similarity

Because semantic textual similarity has many applications in natural language processing, the general form of the problem has attracted a lot of attention from the community [9, 10]. However, it has gained a less but considerable interest regarding the Arabic language. While there are several methods tried to tackle the problem, these methods can be categorized into two main tracks: lexical-based similarity and semantic-based similarity [11]. Lexical-based similarity relies on calculating the correlation between the character streams of two sentences to be compared. This process can be applied to the level of characters or the level of words. While applying this process to the level of characters is relatively simple, it is not robust enough to extract the real similarity between two sentences. Computing the correlation between two texts based on words is better than character level [12]. Methods for measuring similarity between words are using several distance measures to compute the relevance between two terms [13]. Some examples of these measures are Jaccard distance and Levenshtein distance [14, 15].

### Semantic-based similarity

Semantic-based sentence similarity methods can be divided into three classes: word-based sentence similarity, structure-based sentence similarity, and vector-based sentence similarity methods [13]. In word-based sentence similarity, the sentence is handled as a list of words, and the correlation between the words in the two sentences is compared [16]. In structure-based sentence similarity, several methods have been suggested that use language grammar [17], Part-Of-Speech (POS) [18] and words order [19]. Vector-based sentence similarity methods rely on calculating sentence embeddings that describe each sentence as a mathematical vector. These methods are based on corpus analysis. The vector representing each sentence can be calculated by training a model using a sufficiently large corpus. Many techniques have been presented to provide sentence embeddings. For example, Kiros et al. in [20] proposed a method named Skip-Thought that trains an encoder-decoder framework to try to predict the surrounding sentences. In [21], the authors proposed a method that uses siamese transformers and siamese DAN networks to generate sentence embeddings. Cer et al. [22] proposed Universal Sentence Encoder which used unsupervised learning with a transformer network. Conneau et al. [23] proposed InferSent, a siamese BiLSTM network with max-pooling over the output. This method used labeled data of Stanford Natural Language Inference dataset (SNLI) [24] and the MultiGenre NLI dataset (MultiNLI) [25].

### BERT embeddings

**Fig 1:**
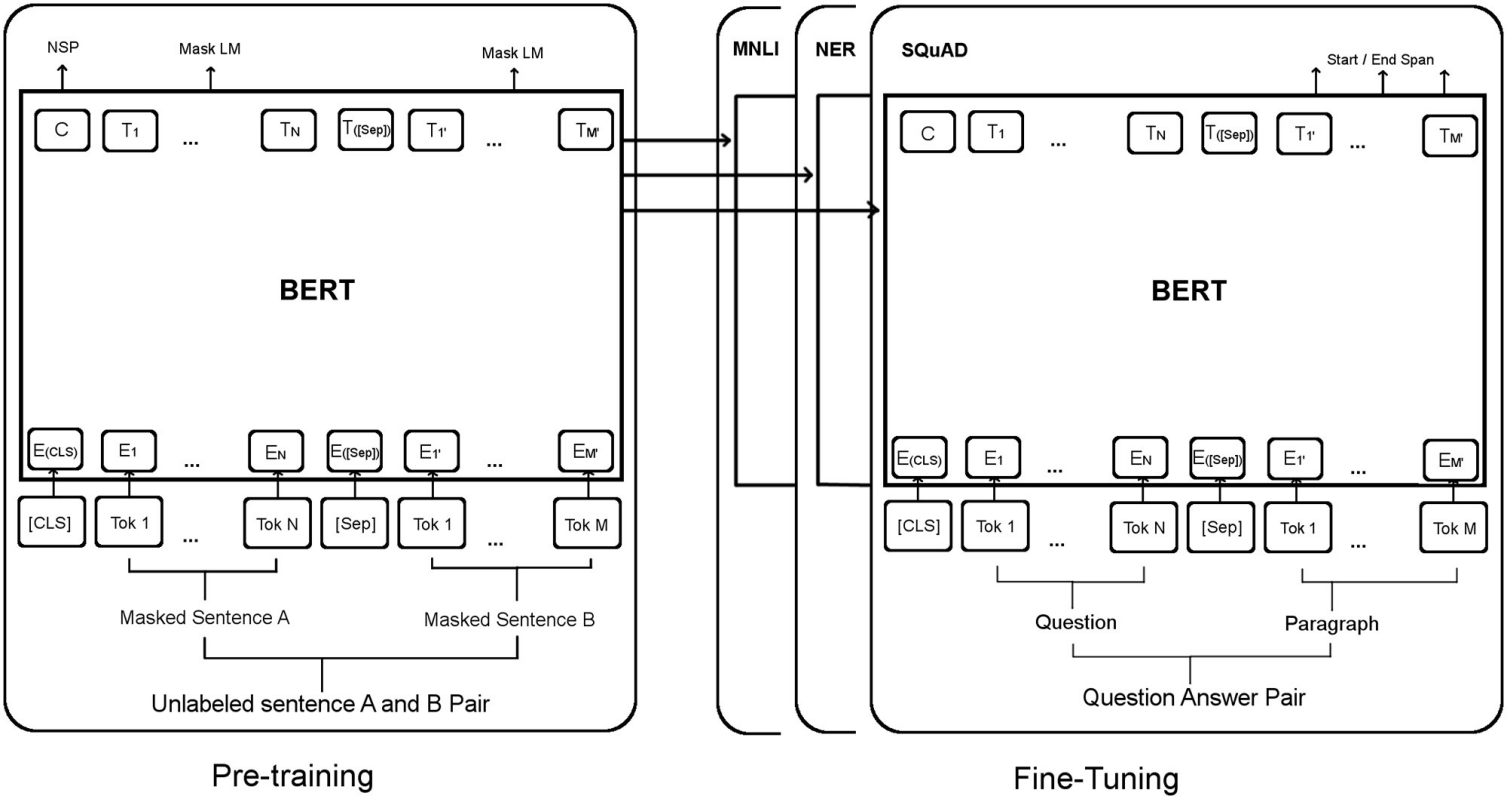
The main framework of BERT.

The main recent approaches to calculate sentence embeddings are based on utilizing robust language models such as BERT. BERT (Fig 1), which stands for Bidirectional Encoder Representations from Transformers, is designed to train masked language models from an unlabeled text by conditioning on both left and right contexts in all layers of a transformer network. Such a language model randomly masks a specific percentage of input tokens and the objective of the training is to predict the original masked tokens using only their context. BERT-based models can be used to generate sentence embeddings. There are several ways to utilize BERT for generating sentence embeddings. For example, by averaging the BERT output layer which is known as BERT embeddings, or by using the embedding of a special token the BERT uses as the first token for each input sentence (Known as the [CLS] token). Also, The BERT can be used in a sentence-pair regression mode to generate a similarity score. However, the embeddings generated by these methods are either not of good quality or not practical for most applications [7].

**Fig 2:**
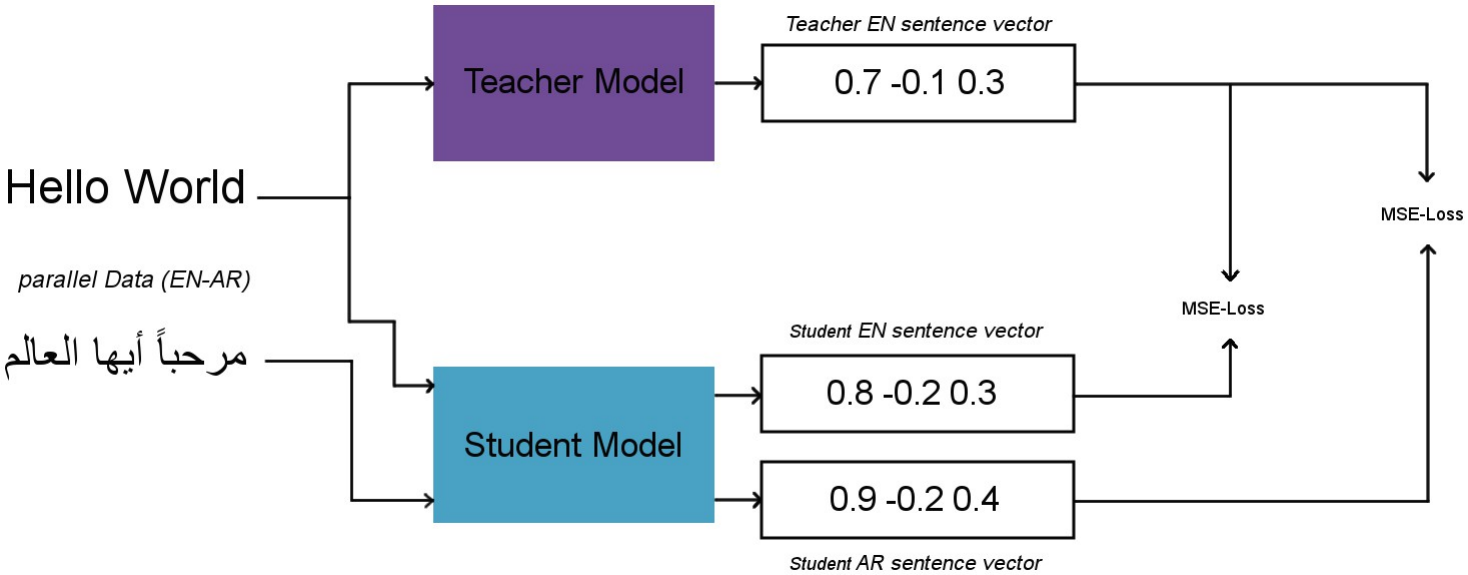
Given parallel data from two languages, a student model can be trained such that the generated vectors for the two languages sentences are close to the teacher language sentence vector.

Several techniques have been proposed to enhance the accuracy of BERT-based sentence embeddings. For example, in [7], the authors present Sentence-BERT (SBERT). The SBERT model [7] takes as input a pair of sentences into siamese architecture which consists of two instances of a base model. Each instance produces an embedding using a pooling procedure. The two embeddings are compared and the manual estimated correlation scores are used to train the model for being oriented to the semantic similarity. In the testing phase, the testing pairs of sentences are given as input to the same architecture and produce a cosine similarity value for each pair of sentences that can be compared with the manual given reference correlation score. The SBERT has been shown to achieve state-of-art performance for the English language STS tasks. To transfer such good performance to other languages, especially those with limited resources, a knowledge distillation approach was proposed [26]. In [26], the authors proposed an efficient method to extend existing sentence embedding models to new languages. Network learning is based on the concept that the original sentence and translated sentences should be mapped in the same location in the vector space. Given, for example, a teacher model of English, they presented an approach to train a student model of another language. They use the original teacher model to produce sentence embeddings for the source language and train a new system using translated sentences to simulate the original model. Fig 2 illustrates an overview of the method. However, using such a technique needs considerable amounts of parallel data from multiple languages to be effective.

### The state-of-the-art

Transfer learning-based solutions for STS have been used in several recent studies. For example, in [27], the authors presented an STS system based on transfer learning. They used an approach that is utilizing RoBERTa [28] models and applied their work to a biomedical dataset. Their proposed methodology obtained an accuracy of 0.9. However, this accuracy was based on domain-specific data. Also in [29], Mutinda et. al. proposed Japanese BERT-based models for textual similarity. They also created two datasets that targeted the clinical medical domain to test their presented systems. They achieved a score of 0.904 on the clinical domain dataset. Furthermore, Yang et. al, in [30] explored 3 transformer-based models for clinical STS, BERT, XLNet [31] and, RoBERTa. They examined transformer models pre-trained using both clinical text and general English text. Their best-performing system was based on a RoBERTa model and obtained a Pearson correlation of 0.9065. However, such good results were due to applying the system to a domain-specific dataset.

Some techniques have been presented to handle the Arabic STS problem. In [26], the authors applied their knowledge distillation-based model on a standard Arabic dataset for testing proposed by [8] and got 79.1 based on Spearman rank correlation. Also, in [32] Nagoudi and Schwab proposed a combination of word embedding and word alignment techniques and then calculated sentence embedding as a sum of its content of word vectors to tackle the Arabic STS problem [9]. Also in [33], Nagoudi et al. proposed a sentence vectors-based method for the cross-lingual similarity between Arabic and English sentences. and they found that using weighting based on POS can enhance their output results.

## Proposed datasets and approaches

### Data

In [8], the authors presented the evaluation of their organized task for Multilingual STS. They have proposed datasets for being used to train and test STS proposed models. The datasets are formatted into pairs of sentences. For each pair, there is a given manual score that indicates the correlation between the two sentences. This score is ranging from 0 (no correlation) to 5 (exact meaning). Table 1 provides some examples of various degrees of correlation between each pair of sentences in the STS datasets.

**Table 1:** Examples of different levels of correlation between the sentences in STS dataset.

Correlation	Example
**5**	**The two sentences have the exact same meaning**
	I don’t see why there should be any problem with this whatsoever.
	I don’t see why that should be a problem.
**4**	**Some unimportant details are different but the two sentences are almost the same**
	A black and white photo of a man driving a car and someone with a motorcycle.
	A black and white photo of a man in a classic car and a man with a classic motorcycle.
**3**	**The two sentences are roughly equivalent, but there are some important different details**.
	A woman is talking on a cell phone.
	A man and woman are talking on the phone.
**2**	**The two sentences are not the same but they share some of the details**
	A man is playing the piano.
	A man played the guitar.
**1**	**The two sentences share the same topic but they are not equivalent**.
	A person is slicing some onions.
	A woman is chopping herbs.
**0**	**The two sentences are completely different**
	The train heads down the tracks and along the hedge.
	A dog on the floor of a patio looks at a cat on the fence.

While Arabic STS was one of their organized tracks, the authors of [8] have provided an MSA Arabic dataset for training, This work adds to them a translation of another 1379 pairs of sentences from the English STS data. The translation has been completed by professional experts. A translation for the same dataset to Egyptian Arabic and Saudi Arabic variants has also been provided by this work. A dataset for testing has been presented in [8]. It consists of 250 pairs of sentences of MSA Arabic. The structure of the testing dataset and training dataset is similar. We proposed a translation of this testing dataset to Egyptian Arabic and Saudi Arabic to be used in evaluation. It is worth mentioning that the testing dataset presented by [8] is a standard measure that is used by state-of-the-art papers (for example [26]). Table 2 illustrates some examples of the proposed translations along with their original English texts.

**Table 2:** Some examples of the proposed translations along with original English sentences.

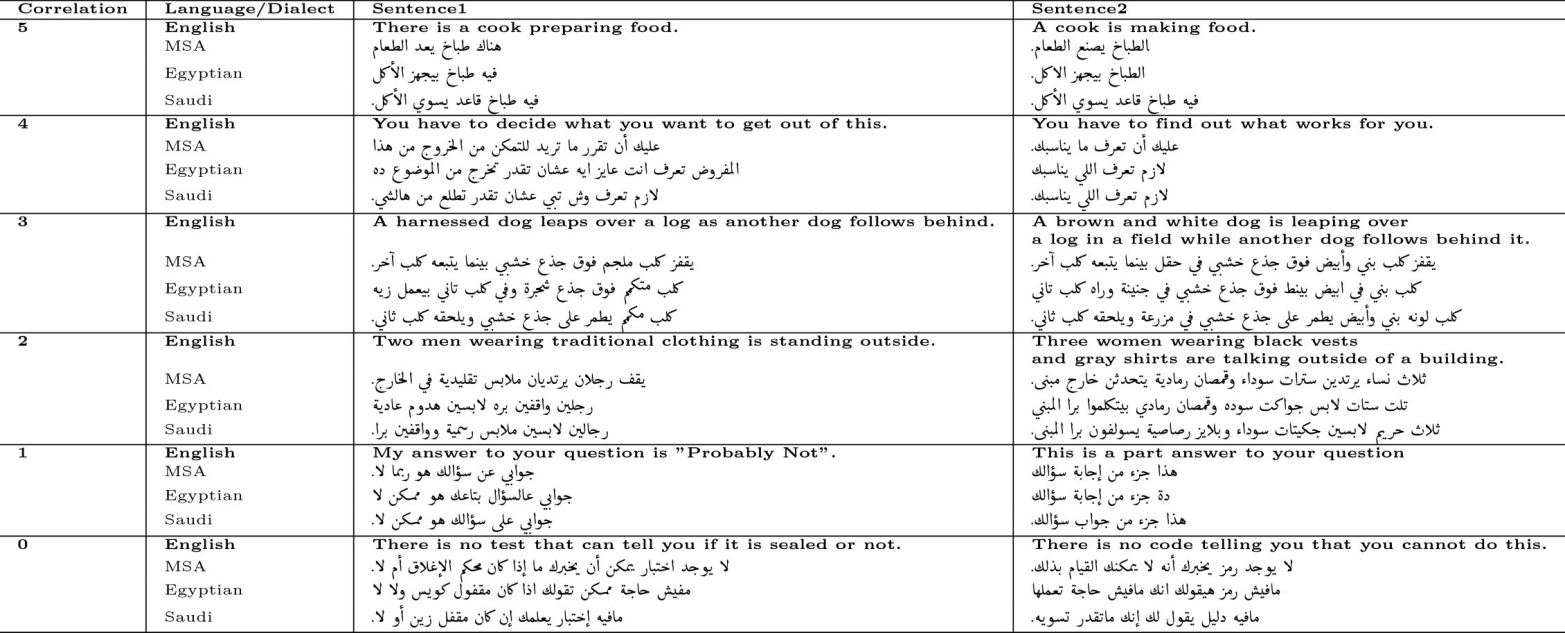

### Methodology

**Fig 3:**
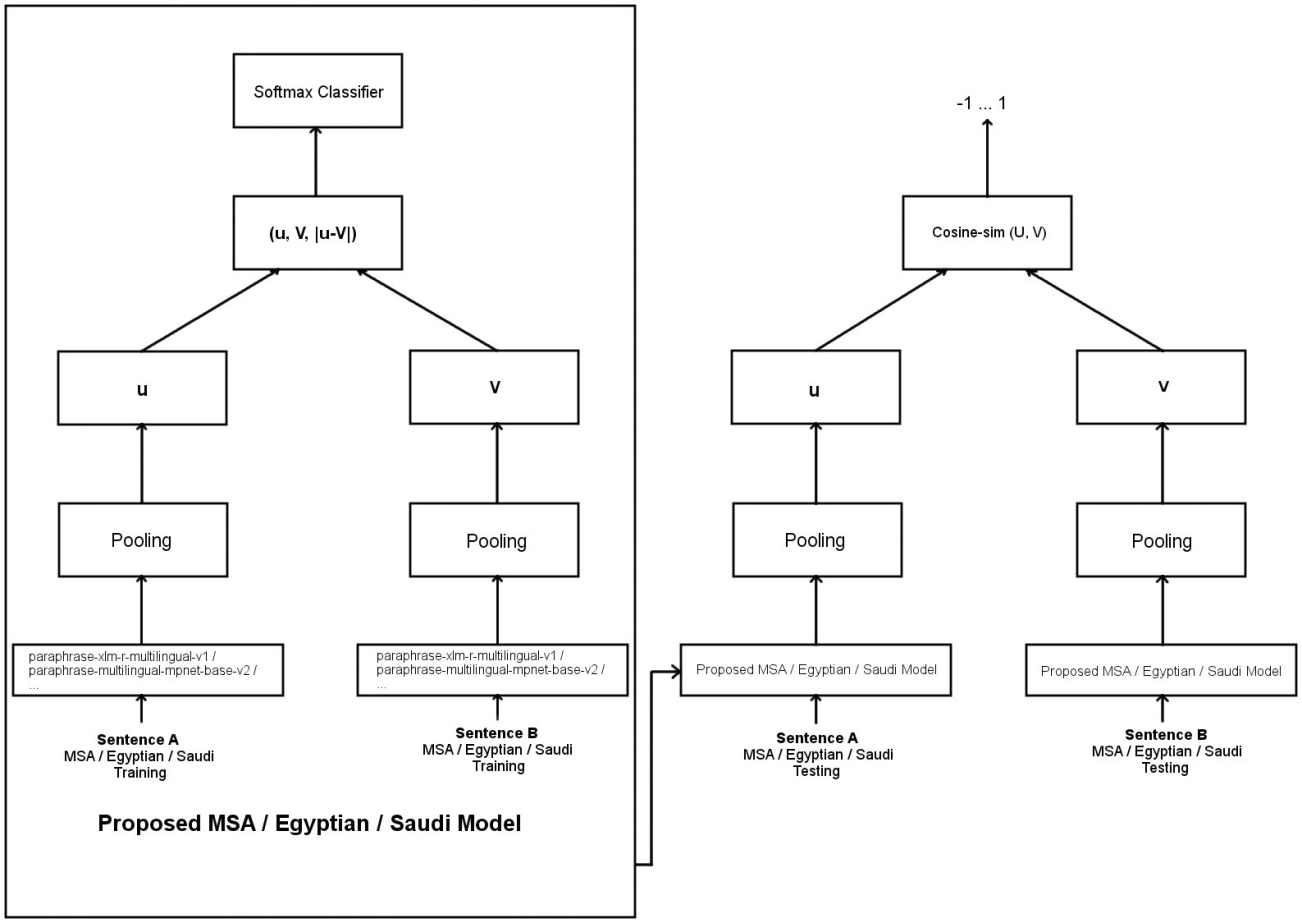
A framework of models generation using the third approach.

To develop our Arabic STS models, three approaches have been used. The first one is to train an SBERT-based model. Such a model is based on an Arabic BERT model that is converted to SBERT structure and fine-tuned using automatic translation to Arabic of the SNLI [24] and MultiNLI [25] English data sets. The M2M100 Many-to-Many multilingual model proposed by [34] has been used for automatic translation of SNLI and MultiNLI datasets to MSA. To build the SBERT-based model, the translated data have been used to convert the ArabicBERT model into an SBERT version. The second approach is based on interleaving English STS data with Arabic BERT models using transfer learning. In this approach, we started with an Arabic BERT-based model. This model has been fine-tuned to be converted to an SBERT model. This was done using English data from SNLI and MultiNLI English datasets and from original STS dataset. As will be seen in the Experimental Results section, this approach considerably impoved the accuracy of the model. The third approach is to utilize knowledge distillation-based STS models as a base and fine-tune the models using the proposed translated dataset to increase the accuracy of the models when used for Arabic STS. First, the pairs of sentences in the translated dataset have been inputted into siamese architecture which consists of two instances of a base model. Each instance produces an embedding using a pooling procedure. The two embeddings are compared and the manual estimated correlation scores are used to guide the network to fine-tune the model for being oriented to the dialect of the input data. Second, in the testing phase, each generated model has been verified using a similar architecture that takes the testing pairs of sentences as input and produces a cosine similarity value for each pair of sentences that can be compared with a manual given reference correlation score. Fig 3 summarizes the framework used in the third approach. The details of implemented experiments are explained in the following section.

## Experimental results

The proposed models have been tested on a standard dataset for testing proposed by [8]. As mentioned before, this dataset has been translated to Egyptian and Saudi Arabic by native speakers of both dialects. Three groups of tests have been applied. In the first group, the accuracy of MSA models has been checked. While in the second and third groups, the generated models of Egyptian and Saudi Arabic have been tested. The accuracy measure that has been used is the standard Spearman rank correlation between the cosine similarity of sentence representations and reference labels of testing datasets. The following is a brief description of the current state-of-the-art STS models and the base models that have been utilized in our experiments:

- **ArabicBERT**: ArabicBERT was the first pre-trained BERT model for Arabic. It is proposed by Safaya et al. in [35].
- **ARBERT**: proposed by Abdul-Mageed et al. in [36]. It is an Arabic large scale masked language model that targets modern standard Arabic.
- **stsb-xlm-r-multilingual**: It is a natural language processing model implemented in Transformer library. It was trained on SNLI + MultiNLI and on STS benchmark dataset. The model is a multilingual version, trained on parallel data for 50+ languages [26].
- **distiluse-base-multilingual-cased-v1**: A multilingual knowledge distilled version of multilingual Universal Sentence Encoder. Supports 15 languages including Arabic and English [26].
- **distiluse-base-multilingual-cased-v2**: It is a multilingual knowledge distilled version of multilingual Universal Sentence Encoder. While v1 model supports 15 languages, this version supports 50+ languages. However, performance on the 15 languages mentioned above are reported to be a bit lower [26].
- **quora-distilbert-multilingual**: It is the multilingual version of quora-distilbert-base, fine-tuned with parallel data for 50+ languages [26].
- **paraphrase-xlm-r-multilingual-v1**: A multilingual version of paraphrase-distilroberta-base-v1, trained on parallel data for 50+ languages [26].
- **paraphrase-multilingual-mpnet-base-v2**: It is the multilingual version of paraphrase-mpnet-base-v2, trained on parallel data for 50+ languages [26].

The following tables show the results of MSA, Egyptian Arabic, and Saudi Arabic experiments respectively. For each table, the base model, the training/fine-tuning data, and the accuracy measured in Spearman/cosine similarity are shown respectively.

### Approaches evaluation

As can be seen in Table 3, in the first experiment, the first approach has been checked. The M2M100 model has been used to automatically translate SNLI and MultiNLI datasets to MSA. M2M100 is a Many-to-Many multilingual translation model proposed by Facebook that can translate directly between any pair of 100 languages. The translated data have been used to convert the ArabicBERT model into an SBERT model. As illustrated in Table 3, when the translated version of SNLI and MultiNLI has been used, the spearman score was around 0.48. But when the original English versions of SNLI and MultiNLI have been used to build the SBERT model, the spearman score was over 0.65. This means that the accuracy achieved using the original SNLI and MultiNLI English version is better than the accuracy we got using the translated version. This may be due to the inaccuracies in the translated version. So, It is not recommended to use automatic language translation-based solutions to tackle the STS problem; at least with the current maturity level of automatic translation.

**Table 3:** Accuracy of machine translation based and interleaved MSA models tested based on Spearman rank correlation between the cosine similarity of sentence representations and the reference labels of the testing dataset in [8].

Base Model	Training data	Score
ArabicBERT bert-base	SNLI and MultiNLI datasets translated using M2M100 model into MSA	0.4798
ArabicBERT bert-base	SNLI and MultiNLI English datasets	0.6525
ARBERT	SNLI and MultiNLI English datasets	0.708
ARBERT	SNLI and MultiNLI English datasets then STS for 1 epoch	**0.7364**

To check the second approach, another experiment has been conducted. We have started with the ARBERT model, which is an Arabic BERT-based model, and fine-tuned it using English data to convert it into an SBERT model, In this direction, two trials have been tested, in the first trial, only SNLI and MultiNLI English datasets have been used for model conversion. while in the other trial, SNLI and MultiNLI datasets have been utilized and then a finetuning process has been applied using original STS data [8] for one epoch. The first trial provided a spearman score of around 0.70 while with the second trial, we got an accuracy of over 0.73. From these two trials, It can be seen that interleaving English data with Arabic-based models is more promising than the translation-based solution.

In the third approach, It has been checked how efficiently to use knowledge distillation-based solutions. For this purpose, several experiments have been conducted. As shown in Table 4, our translated 1.3k pairs of sentences have been used to fine-tune several state-of-the-art STS models. The best Spearman score achieved using this approach was over 0.81 using paraphrase-multilingual-mpnet-base-v2 proposed by [26] as a base model and our proposed translated dataset along with original data presented by [8]. Using a similar procedure, the proposed translated versions of STS data to Egyptian Arabic and Saudi Arabic have been used to fine-tune the state-of-the-art models. As illustrated in Table 5, In the case of Egyptian Arabic, the proposed translated data have been successfully used to fine-tune the base model paraphrase-xlm-r-multilingual-v1 proposed by [26] with a Spearman score of 0.775. In the case of Saudi Arabic. the proposed translated Saudi data along with the original data proposed by [8] have been utilized to fine-tune state-of-the-art base models. Table 6 provides the details of the experiments done in this direction. As shown in Table 6, the best Spearman score achieved was over 0.76 by fine-tuning the base model distiluse-base-multilingual-cased-v2.

**Table 4:** Accuracy of knowledge distillation-based MSA models tested based on Spearman rank correlation between the cosine similarity of sentence representations and the reference labels of the testing dataset in [8].

Base Model	Fine-tuning data	Score
quora-distilbert-multilingual	translated 1.3K MSA pairs of sentences	0.7665
distiluse-base-multilingual-cased-v2	translated 1.3K MSA pairs of sentences	0.7752
distiluse-base-multilingual-cased-v1	translated 1.3K MSA pairs of sentences	0.7778
stsb-xlm-r-multilingual	translated 1.3K MSA pairs of sentences	0.7785
paraphrase-xlm-r-multilingual-v1	translated 1.3K MSA pairs of sentences	0.7918
paraphrase-xlm-r-multilingual-v1	translated 1.3K MSA pairs of sentences + original Arabic STS	0.7999
paraphrase-multilingual-mpnet-base-v2	translated 1.3K MSA pairs of sentences	0.8012
paraphrase-multilingual-mpnet-base-v2	translated 1.3K MSA pairs of sentences + original Arabic STS	**0.8103**

**Table 5:** Accuracy of main Egyptian models tested based on Spearman rank correlation between the cosine similarity of sentence representations and the reference labels of the testing dataset in [8] after translation to Egyptian Arabic.

Base Model	Fine-tuning data	Score
paraphrase-multilg-mpnet-base-v2	translated 1.3K Egyptian pairs of sentences	0.7345
paraphrase-multilg-mpnet-base-v2	original Arabic STS then the translated 1.3K Egyptian pairs of sentences	0.763
paraphrase-xlm-r-multilingual-v1	original Arabic STS then the translated 1.3K Egyptian pairs of sentences	0.7647
paraphrase-xlm-r-multilingual-v1	translated 1.3K Egyptian pairs of sentences	**0.7751**

**Table 6:** Accuracy of main Saudi Arabian models based on Spearman rank correlation between the cosine similarity of sentence representations and the reference labels of the testing dataset in [8] after translation to Saudi Arabic.

Base Model	Fine-tuning data	Score
paraphrase-xlm-r-multilingual-v1	translated 1.3K Saudi pairs of sentences	0.7441
paraphrase-xlm-r-multilingual-v1	original Arabic STS then the translated 1.3K Saudi pairs of sentences	0.752
distiluse-base-multilingual-cased-v2	translated 1.3K Saudi pairs of sentences	0.7608
distiluse-base-multilingual-cased-v2	original Arabic STS then the translated 1.3K Saudi pairs of sentences	**0.7622**

## Comparisons with state-of-the-art

To test the quality of the proposed models, they have been compared to state-of-the-art counterparts. While different methods have been assessed on various datasets at testing, our results can be compared to methods that used the MSA testing dataset suggested in [8]. Table 7 illustrates the comparisons with the best current MSA models.

**Table 7:** Comparisons between the proposed models and current state-of-the-art Arabic STS models based on Spearman rank correlation between the cosine similarity of sentence representations and the reference labels of the testing dataset in [8].

Variant	Model	Spearman/Cosine similarity
**MSA**	quora-distilbert-multilingual	0.7075
	distiluse-base-multilingual-cased-v1	0.7586
	distiluse-base-multilingual-cased-v2	0.7734
	stsb-xlm-r-multilingual	0.7867
	paraphrase-xlm-r-multilingual-v1	0.791
	paraphrase-multilingual-mpnet-base-v2	0.791
	proposed MSA model	**0.8103**
**Egyptian**	**Model**	**Spearman/Cosine similarity**
	quora-distilbert-multilingual	0.5811
	paraphrase-multilingual-mpnet-base-v2	0.6847
	distiluse-base-multilingual-cased-v2	0.6950
	stsb-xlm-r-multilingual	0.7200
	distiluse-base-multilingual-cased-v1	0.7237
	paraphrase-xlm-r-multilingual-v1	0.7516
	proposed Egyptian model	**0.7751**
**Saudi**	**Model**	**Spearman/Cosine similarity**
	quora-distilbert-multilingual	0.5706
	paraphrase-multilingual-mpnet-base-v2	0.6784
	stsb-xlm-r-multilingual	0.6879
	paraphrase-xlm-r-multilingual-v1	0.7145
	distiluse-base-multilingual-cased-v1	0.7310
	distiluse-base-multilingual-cased-v2	0.7410
	proposed Saudi model	**0.7622**

As shown in Table 7, the proposed model for MSA enhanced the state-of-the-art result by around an absolute 2%. It is worth mentioning that transfer learning-based solutions depend on the similarity between the domain of the base model and the domain of the new model. While the base model (paraphrase-multilingual-mpnet-base-v2) of the proposed MSA model has been trained on large scale amounts of data [26], the proposed new model has been fine-tuned using small dataset of only a few thousands of sentence pairs. This is promising because it indicates that the results can be even improved more without a need for new large scale datasets.

While there are no models in the literature that intentionally target Egyptian and Saudi Arabic, the state-of-the-art multilingual model that supports MSA Arabic provides a good result for the Egyptian Arabic variant. But the contributed model that targets the Egyptian Arabic boosts the result by 2.4% absolute enhancement. And the proposed Saudi-focused model also provided around 2% absolute gain. However, the gap between the accuracy achieved in MSA versus the Egyptian and Saudi dialects is still considerable. This is largely because the base model used has been trained on MSA data, while the Egyptian and Saudi variants didn’t appear in the training data of their base models. To tackle this problem in the future, is it planned to automatically extract parallel data of high quality between MSA and Egyptian Arabic and between MSA and Saudi Arabic. And then using these data to boost the performance of Egyptian and Saudi models to match the level of MSA.

## Discussions and conclusions

In this paper, the semantic textual similarity problem has been addressed with a focus on the Arabic language and two of the major Arabic dialectical variants: Egyptian and Saudi Arabic. The Arabic language is one of the low-resourced languages. This produces a considerable lag of accuracy between semantic textual similarity models of Arabic and their counterparts in rich-resourced languages such as English. The suggested work has been presented to tackle this problem. The main contributions proposed in the paper can be summarized in the following: First, the problem of limited resources for Arabic STS has been addressed by three approaches. The first approach is to utilize automatic machine translation to translate English STS data to Arabic and to use the translated data for converting Arabic BERT models into STS Arabic models. The second approach is to interleave English STS data with Arabic BERT models to produce improved Arabic STS models. The third approach is based on utilizing knowledge distillation-based models as a base and fine-tuning them using a proposed translated dataset to improve the performance for Arabic STS. Also, we contributed a manual translation of a large subset from the STS competition dataset [8]. It has been translated to modern standard Arabic, Egyptian Arabic, and Saudi Arabic by professional translators. Moreover, the developed models that enhanced the accuracy for modern standard Arabic STS by around absolute 2% gain over the state-of-the-art level have been presented. The models have been tested on the standard dataset used by the community. Furthermore, the work presented the details and experiments of the developed STS models for Egyptian Arabic and Saudi Arabic, which achieved gains of around absolute 2.4% and 2% respectively.

Based on these results, the main conclusions to be considered are the following: Delivering high-quality data to the community is of special importance to improve the accuracy of STS models of low-resourced languages such as Arabic. Also, knowledge distillation based solutions are competitive to tackle the STS problem. Furthermore, the accuracy of Egyptian Arabic and Saudi Arabic STS models can be boosted considerably even with using relatively small proposed datasets.

### Limitations and future work

Although the suggested work presents significant improvement for Arabic MSA STS, there are still some limitations to be considered. First, there is a large gap between the accuracy of MSA STS models when compared with the state-of-the-art of English STS. It is important to minimize this gap to support models integration in practical applications. Also, evaluation measures should consider the embedded semantic information included in sentences such as named entities. Our future work plan is to check the robustness of the developed Arabic STS models by evaluating them using different downstream tasks such as question answering and Quora Question Pairs problem [37]. Moreover, it is planned to expand our work by targeting new important Arabic dialects such as Maghribi and Levantine variants.

## Supporting information

S1 Data (ZIP)
